# Treatment of Refractory High-Flow Chylothorax in High-Grade B-Cell Lymphoma by Intratumoral Lymphatic Embolization

**DOI:** 10.1007/s00270-021-02931-0

**Published:** 2021-08-05

**Authors:** Florian Streitparth, Sebastian Theurich, Tina Streitparth, Osman Öcal, David Cordas dos Santos, Wilhelm Flatz

**Affiliations:** 1 Department of Radiology, University Hospital, Ludwig-Maximilians University (LMU), Marchioninistr. 15, 81377 Munich, Germany; 2 Department III of Internal Medicine, University Hospital, LMU, Munich, Germany; 3 Cancer- and Immunometabolism Research Group, Gene Center, LMU, Munich, Germany; 4 German Cancer Research Center (DKFZ), LMU University Hospital Munich, German Cancer Consortium (DKTK), Partner Site Munich, Heidelberg, Germany; 5 Comprehensive Cancer Center Munich, LMU, Munich, Germany

**Keywords:** Non-Hodgkin lymphoma, Intratumoral lymphography, Embolization, Chylothorax, Wasting syndrome

To the Editor,

Chylous effusion is a rare and potentially fatal complication of lymphoma due to respiratory complications and nutritional wasting syndrome [1]. In refractory chyle leaks, lymphography and lymphatic embolization can identify and occlude lymphatic leakages [2–4]. However, intratumoral lymphography has not been reported so far. Here, we report intratumoral lymphography and embolization in the treatment of lymphoma-related refractory high-flow chylothorax.

A 69-year-old female presented with mediastinal and abdominal/retroperitoneal lymph bulks and bilateral pleural effusions leading to severe dyspnea (Fig. 1a). Biopsy revealed follicular B-cell Non-Hodgkin lymphoma with partially transformation into a high-grade lymphoma. Pleural drainage revealed a milky pleural effusion of 4500 mL/day with elevated triglyceride levels (3414 mg/dL) and high positivity of chylomicrons (2/3), consistent with chyle.

**Fig. 1:**
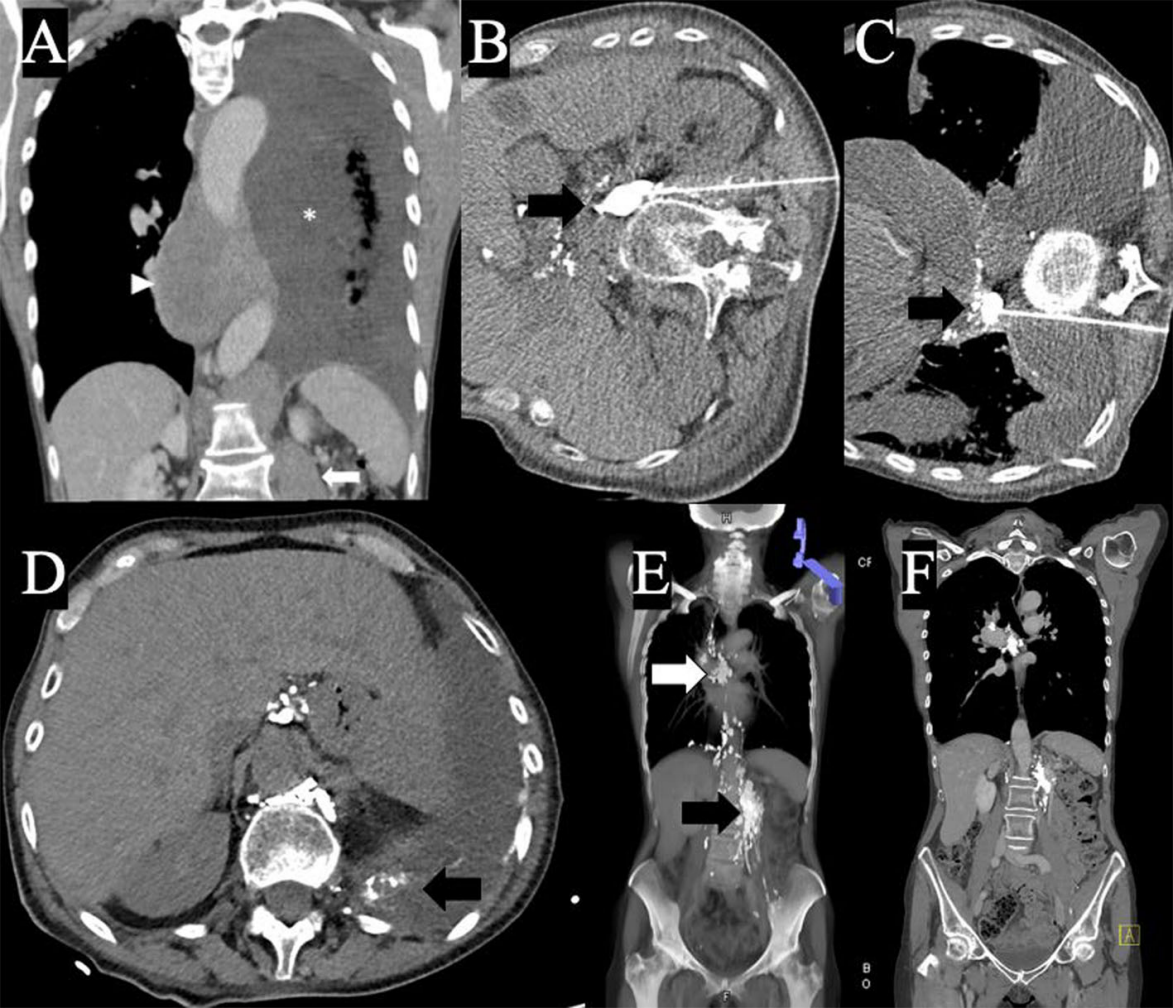
**A** Pretreatment coronal contrast-enhanced CT image shows thoracic (arrowhead) and abdominal (arrow) large lymphoma bulks and pleural effusion (asterisk). CT-fluoroscopic image shows canulation of **B** abdominal and **C** mediastinal lymphoma bulk (arrow) percutaneously with a needle and collection of injected lipiodol in bulk **D** control scan at the end of the procedure showed lipiodol-glue accumulation within bilateral pleural effusion (arrow, right side not shown) **E** coronal maximum intensity projection image two weeks after the procedure shows no pleural effusion. Note the residual lipiodol depositions within the shrunken lymphoma bulks (arrows) and lymph vessels in which lipiodol transported antegrade **F** 1-year follow-up CT image shows complete remission of the lymphoma with no pleural effusion while still showing residual lipiodol deposition

Medium-chain triglycerides-based diet and parenteral nutrition failed to reduce drainage. Intravenous human albumin replacement was necessary due to protein-loss syndrome with severe anasarca and marasmus. A systemic immunochemotherapy with R-CHOP-14 was initiated, and CT staging after two cycles revealed an overall good response. However, the high-output chylothorax was unchanged.

Lymphography and lymph-embolization were decided after interdisciplinary discussion. After informed consent, under analgosedation and local anesthesia, the retroperitoneal lymph bulk was punctured with a 20G Chiba-needle under CT guidance (Fig. 1b), and a total of 12 mL (5 mL/h) lipiodol (Guerbet GmbH, Sulzbach, Germany) was injected according to our prospective study protocol INTACT-lymph (Protocol No. DRKS00015299, German Clinical Trials Register). The post-procedural CT showed marked lipiodol opacification of the lymphoma bulks with mainly anterograde lipiodol distribution and lipiodol leakage into the chylous effusions in both sides. Since drainage was not sufficiently reduced, a CT-guided lymph-embolization was performed through the same retroperitoneal puncture site and an additional lipiodol-tagged mediastinal bulk 13 days later with an injection of 10 mL lipiodol and n-Butyl-2-cyanoacrylate (n-BCA glue; Histoacryl, B. Braun, Melsungen, Germany) in a mixture of 5:1, simultaneously 5 mL in each side (Fig. 2).

**Fig. 2:**
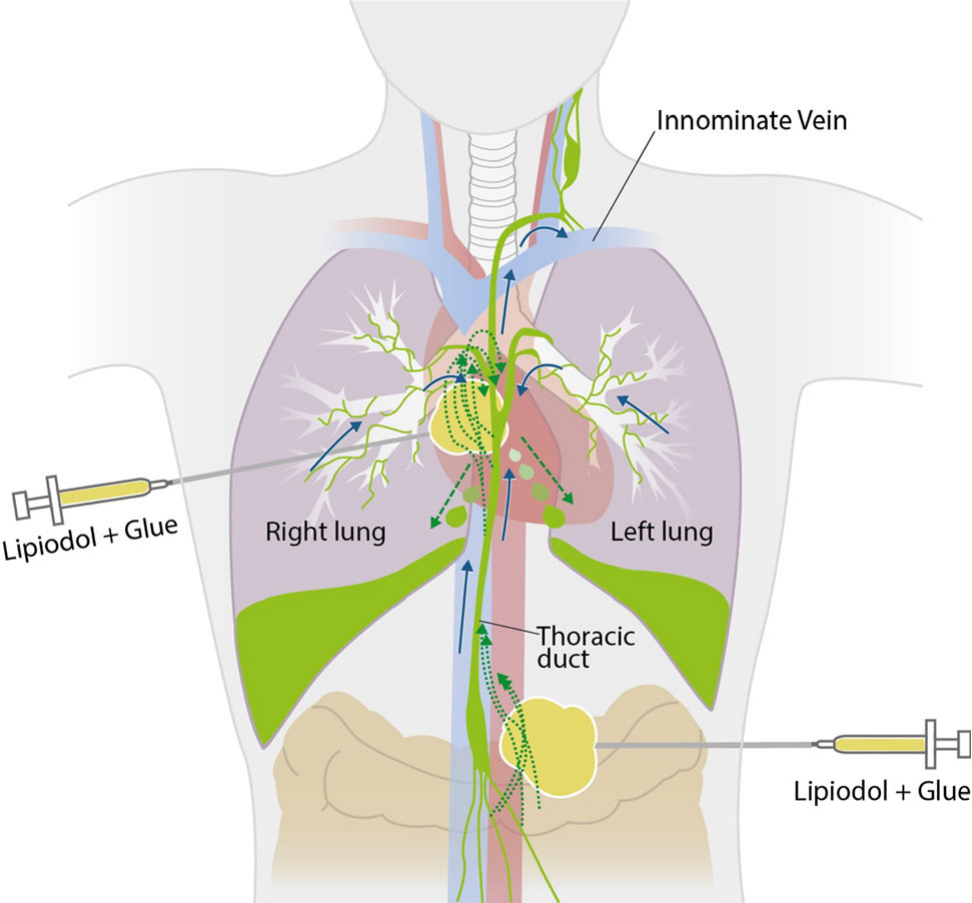
Blue arrows indicate normal lymphatic flow. Chaotic and disrupted lymphatic channels (dotted green arrows) within lymphoma bulks lead to abnormal lymph accumulation (dashed green arrow) and chylothorax. Puncture of the lymphoma bulks under CT guidance and injection of the lipiodol-glue mixture

Drainage reduced immediately and pleural catheter was removed ten days after. The clinical situation improved and control CT scans before discharge showed no pleural effusions (Fig. 1c). Post-interventional ECOG-PS increased during hospitalization from 4 to 2, and to 0 at 3-month follow-up. Control CT scans showed no chylothorax and complete remission of the lymphoma (Fig. 1d).

Chylous effusions can be seen due to iatrogenic or accidental trauma and inflammatory or malignant diseases, specifically lymphoma [1, 5]. Lymphoma-associated chylous effusion can result from impaired lymphatic drainage or invasion of lymphatics [1]. Chylous leaks may resolve with systemic lymphoma treatment [5]. However, high-output chylothorax cases may persist and lead wasting syndrome which can become life-threatening and difficult to manage. If leakages persist under systemic therapy, diet and parenteral nutrition can reduce chyle flow, and pharmacological treatment with octreotide, surgery, or low-dose radiotherapy are alternative options. However, the success rates of the mentioned treatments are limited.

Lymphography and lymphatic embolization are promising options if conservative treatment is unsuccessful. Lymphography with lipiodol was shown to detect and also embolize lymphatic leakage in 51–75% of patients [2–4]. However, the success of lipiodol alone drops from 70 to 35% in cases with high-output drainage [2]. As an additional treatment option, injection of glue or sclerotic agents is used for definitive leak embolization (3). However, in malignancies, lymphatic vessels are disorganized within the tumor tissue and thus might prevent conventional lymphography. In our patient, systemic therapy led to a rapid shrinkage of the lymphoma manifestations, and ultimately, complete remission. Despite tumor response, chylothorax persisted. This report describes a case of CT-guided intratumoral lymph-embolization for lymphoma-associated chylothorax. Reduction of chylothorax was imminent and permanent after combined injection of lipiodol and n-BCA glue into the lymphoma bulks.

In conclusion, intratumoral lipiodol lymphography and adjunctive glue embolization could serve as an effective minimal-invasive treatment option with possible long-term remission in lymphoma-associated chylothorax.

## References

[1] Bhardwaj R, Vaziri H, Gautam A, Ballesteros E, Karimeddini D, Wu GY (2018). Chylous ascites: a review of pathogenesis, diagnosis and treatment. J Clin Transl Hepatol.

[2] Alejandre-Lafont E, Krompiec C, Rau WS, Krombach GA (2011). Effectiveness of therapeutic lymphography on lymphatic leakage. Acta Radiol.

[3] Kortes N, Radeleff B, Sommer CM, Bellemann N, Ott K, Richter GM (2014). Therapeutic lymphangiography and CT-guided sclerotherapy for the treatment of refractory lymphatic leakage. J Vasc Interv Radiol.

[4] Nadolski GJ, Chauhan NR, Itkin M (2018). Lymphangiography and Lymphatic Embolization for the Treatment of Refractory Chylous Ascites. Cardiovasc Intervent Radiol.

[5] Kako S, Joshita S, Matsuo A, Kawaguchi K, Umemura T, Tanaka E (2019). A Case of Adult T-Cell Leukemia/Lymphoma Complicated with Bilateral Chylothorax. Case Rep Oncol Med.

